# Acute Neonatal Infections ‘Lock-In’ a Suboptimal CD8+ T Cell Repertoire with Impaired Recall Responses

**DOI:** 10.1371/journal.ppat.1003572

**Published:** 2013-09-12

**Authors:** Brian D. Rudd, Vanessa Venturi, Norah L. Smith, Kito Nzingha, Emily L. Goldberg, Gang Li, Janko Nikolich-Zugich, Miles P. Davenport

**Affiliations:** 1 Department of Microbiology and Immunology, Cornell University, Ithaca, New York, United States of America; 2 Computational Biology Group, Centre for Vascular Research, University of New South Wales, Kensington, New South Wales, Australia; 3 Department of Immunobiology and the Arizona Center on Aging, University of Arizona College of Medicine, Tucson, Arizona, and the BIO5 Institute, University of Arizona, Tucson, Arizona, United States of America; 4 Complex Systems in Biology Group, Centre for Vascular Research, University of New South Wales, Kensington, New South Wales, Australia; University of Minnesota Medical School, United States of America

## Abstract

Microbial infection during various stages of human development produces widely different clinical outcomes, yet the links between age-related changes in the immune compartment and functional immunity remain unclear. The ability of the immune system to respond to specific antigens and mediate protection in early life is closely correlated with the level of diversification of lymphocyte antigen receptors. We have previously shown that the neonatal primary CD8+ T cell response to replication competent virus is significantly constricted compared to the adult response. In the present study, we have analyzed the subsequent formation of neonatal memory CD8+ T cells and their response to secondary infectious challenge. In particular, we asked whether the less diverse CD8+ T cell clonotypes that are elicited by neonatal vaccination with replication competent virus are ‘locked-in’ to the adult memory T cell, and thus may compromise the strength of adult immunity. Here we report that neonatal memory CD8+ T cells mediate poor recall responses compared to adults and are comprised of a repertoire of lower avidity T cells. During a later infectious challenge the neonatal memory CD8+ T cells compete poorly with the fully diverse repertoire of naïve adult CD8+ T cells and are outgrown by the adult primary response. This has important implications for the timing of vaccination in early life.

## Author Summary

Newborns typically have a heightened sensitivity to infectious diseases, the reasons for which are not yet well understood. One contributing factor is the limited diversity of lymphocyte receptors early in life to recognize antigen and control infection. We have previously shown that antigen-specific CD8+ T cell repertoires are significantly constricted in neonates compared with adults. In this study, we addressed the question of whether the developmental stage of the host at the time of vaccination influences the composition of the memory CD8^+^ T cell repertoire and its ability to mount a robust response to subsequent infections. We observed that the antigen-specific T cell repertoires elicited in the context of an acute neonatal infection, that are less diverse and comprised of lower-avidity T cells, are partially ‘locked-in’ to the adult memory T cell repertoire. However, in the face of a secondary infectious challenge, naïve adult T cells outcompete the lower avidity neonatal memory T cells and raise the diversity of the overall CD8^+^ T cell response. These results have potential implications for the design of vaccines to be administered in early life.

## Introduction

The immune system of neonates is generally characterized as immature and more susceptible to infections with various pathogens [1]–[3]. Many of the most debilitating infections are inflicted by intracellular pathogens that are either vertically transmitted or acquired very early on in life (e.g. HIV, CMV, EBV, TB, HSV). Although CD8+ T cells are considered the key players in combating these intracellular pathogens, their capacity to provide protective immunity in neonates is still poorly understood. Importantly, since the timing of infection in some cases affects the subsequent pathogen load and pathogenesis of infection, we wished to understand whether early exposure to infection or vaccination compromises the later ability to control infection as an adult.

The ability of CD8+ T cells to mount a protective response to new pathogens is dependent upon the presence of a broad repertoire of T cells of appropriate immune functionality [4], [5]. Diversification of the repertoire is developmentally regulated and the neonatal T cell repertoire in mice is restricted not only by the reduced number of T cells that are present, but also by the number of unique antigen receptors that are able to be produced. Diversity of T-cell receptor (TCR) usage is accomplished by multiple mechanisms during T-cell maturation in the thymus [6]. Somatic recombination of germline segments identified as variable (V), diversity (D), and joining (J) segments by Rag-1 and Rag-2 proteins results in an enormous number of T cells with distinct antigen binding domains. Further diversification is accomplished by nibbling or loss of germline-encoded nucleotides and the addition of complementary template-dependent (P) and random template-independent (N) nucleotide additions at the junctions between these germline segments prior to ligation [7]. The addition of N regions between germline-encoded segments is mediated entirely by terminal deoxynucleotidyl transferase (TdT) and it has been estimated that 90–95% of the diversity of the T cell repertoire is attributed to this critical step [8].

The expression of TdT is likely to have a significant impact on both the quantity and quality of TCR clonotypes that are able to respond to various pathogens at different stages of development. In mice, TdT is not upregulated in the thymus until 4–5 days after birth, with significant nucleotide additions being observed at day 8 [9], [10]. Thus, in the first week of life we would still expect much of the peripheral T cell repertoire to be comprised of clonotypes that have not been sculpted by TdT and thus be devoid of N-additions. Indeed, we recently have characterized the TCRβ repertoire of CD8+ T cells responding to the immunodominant HSV-1 epitope, gB_498–505_/K^b^ (gB-8p) in neonate [11] and adult mice [12] and showed that the gB-8p TCRβ repertoire in neonatal mice is severely restricted and comprised of more germline sequence-rich clonotypes [11]. This restricted TCR repertoire in neonates may have direct effects on the ability of primary neonatal CD8+ T cells to respond to antigen, as well as indirect effects on their ability to transition into the long-lived memory pool. The key question we wished to examine in this report is whether the primary CD8+ T cell response in neonates induces a memory pool of sufficient diversity to later mount a robust secondary response to infection, or whether neonatal infections ‘lock-in’ a poor memory CD8+ T cell population that exhibits impaired recall responses in later life. Here, we demonstrate how the developmental stage of the host at the time of vaccination or primary infection can alter the composition of the long-lived memory CD8+ T cell pool, as well as their ability to respond to subsequent infections.

## Results

### Quantification of gB-8p-specific CD8+ T cell responses in neonatal and adult mice

**Figure 1:**
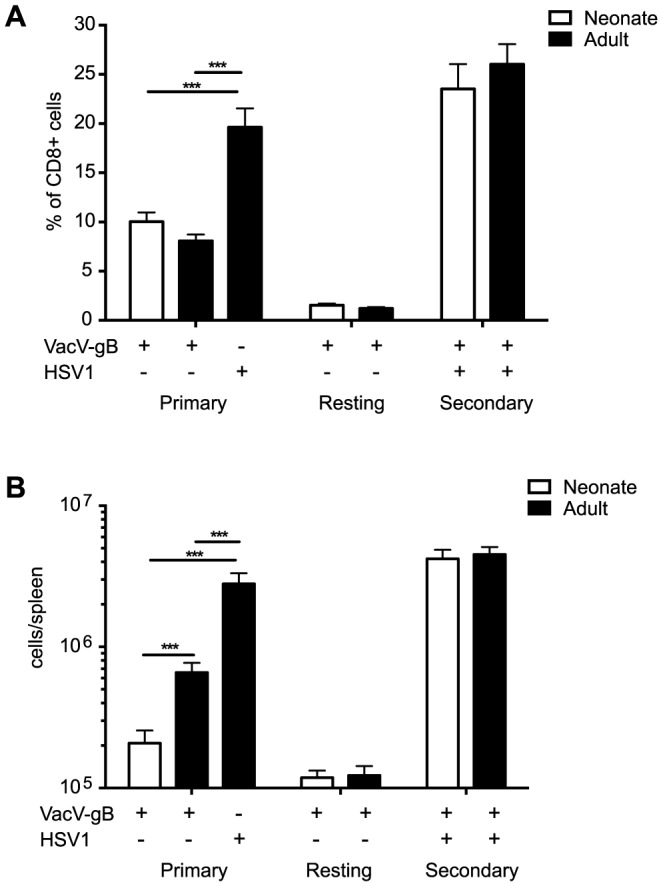
Magnitude of primary and secondary gB-8p-specific CD8+ T cell responses. Neonatal and adult B6 mice were vaccinated with VACV-gB and challenged 6 weeks later with HSV-1. The frequency (A) and total numbers (B) of gB-8p-specific CD8+ T cells were enumerated in the spleen by tetramer staining at the peak of VACV-gB infection (day 6 for adults, day 8 for neonates), at 6 weeks post-vaccination, and at the peak of HSV-1 challenge (day 6). Naive adult B6 mice were also infected with HSV-1 for comparison with the secondary CD8+ T cell responses. All data is representative of 3–4 separate experiments with n = 6–8 mice/group. Results depict mean ± SEM. The data shown for the neonatal and adult CD8+ T cell responses to primary VACV-gB infection were obtained in previous studies [11], [12].

In this report, we aimed to compare antiviral memory CD8+ T cells that were generated in either neonatal or adult stages of development. Over 90% of the CD8+ T cell response in HSV-1-infected C57BL/6 mice is directed against a single K^b^-restricted immunodominant epitope in the glycoprotein B (denoted gB-8p) [13]. To compare the expansion of naïve and memory gB-8p CD8+ T cells in neonatal (7-day old) and adult mice (8–12 week-old), both age groups were acutely infected with vaccinia virus expressing the gB-8p peptide (VACV-gB, i.p.) and challenged 6–8 weeks later with HSV-1 (i.p.). In this way, we were able to mimic neonatal vaccinations with live viral vectors and preferentially prime the neonatal T cells that were available in early life. However, smaller numbers of peripheral T cells are present in neonatal mice compared to adults [14], [15]. Therefore, as in our previous study of the neonatal primary response [11], the dose of VACV-gB was normalized in neonates (2×10^1^ PFU/mouse) and adults (2×10^5^ PFU/mouse) by titrating VACV-gB doses down to the least amount of virus that was required to elicit a comparable relative frequency (∼10%) of gB-8p CD8+ T cells at the peak of the response (Fig. 1A). This difference in viral dose is necessary as an adult dose is lethal to neonates and adult mice administered a neonatal dose clear the virus too rapidly to allow the detection of antigen-specific CD8+ T cells. In addition, as primary vaccinia virus infection is intended to mimic vaccination at different ages, we note that a decreased dose of immunogen is routinely administered to children for a variety of human vaccine protocols. While the total number of gB-8p CD8+ T cells was much higher in adults at the peak of the primary infection (due to increased cellularity), similar levels of gB-8p-specific memory T cells were observed in neonatal- and adult-immunized mice by 6 weeks post-infection (Fig. 1B). All mice were then challenged with 1×10^6^ pfu of HSV-1 (i.p.), and we observed a secondary CD8+ T cell response that was comparable between neonate and adult-vaccinated mice (Fig. 1B).

### Clonotypic composition of the secondary gB-8p-specific TCRβ repertoires in neonatal and adult mice

We recently have compared the clonal composition of the gB-8p-specific TCRβ repertoires involved in the primary CD8^+^ T cell responses to VACV-gB in neonatal and adult mice [11]. The gB-8p-specific TCRβ repertoires in neonatal mice were found to have the same basic features, in terms of gene usage biases and CDR3β amino acid motif, as in adult mice. However, the significantly less diverse gB-8p-specific Vβ10+ TCRβ repertoires of neonatal mice were predominantly comprised of shorter germline-gene-encoded CDR3β sequences. This published data on the primary response to vaccination in adults and neonates was used as a baseline for comparison with the secondary responses following challenge.

**Figure 2:**
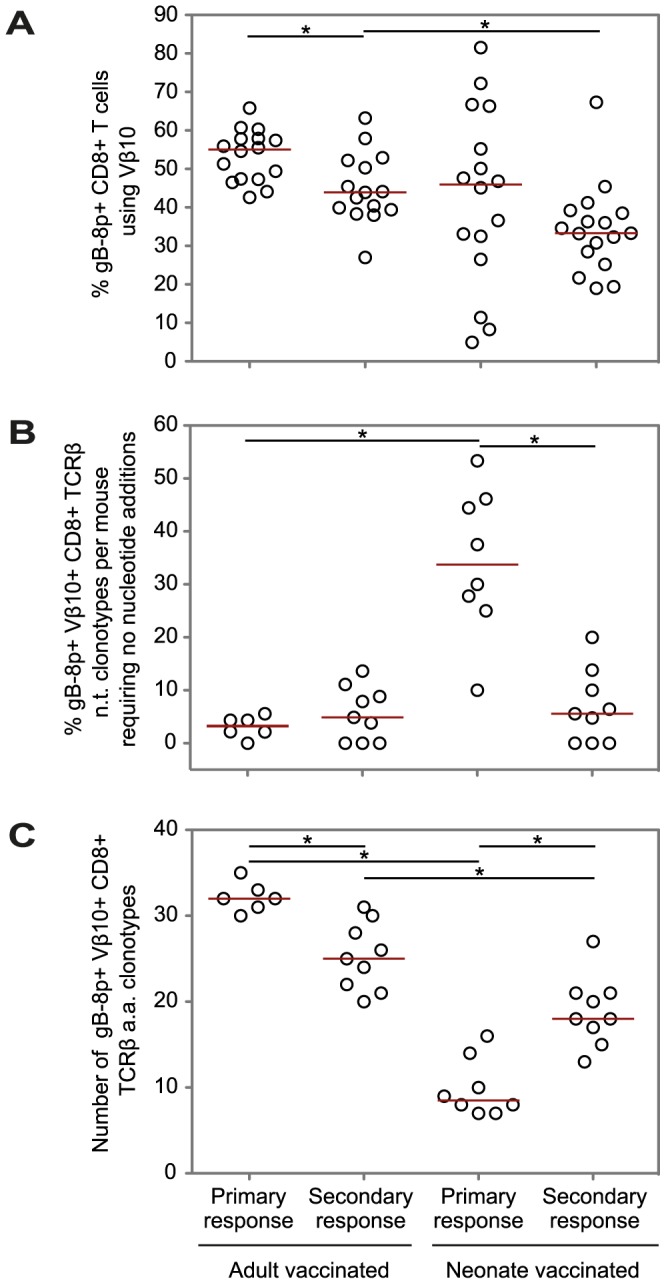
Comparison of the features of the gB-8p-specific CD8+ TCRβ repertoires for primary and secondary responses. The features of the gB-8p-specific TCRβ repertoires involved in the primary responses to VACV-gB in neonatal and adult mice and the secondary responses to HSV-1 in neonatal-vaccinated and adult-vaccinated mice. Shown are the percentage of gB-8p-specific CD8+ T cells per mouse that use the Vβ10 gene (A); the percentage of unique gB-8p-specific Vβ10+ TCRβ nucleotide (n.t.) clonotypes per mouse requiring no nucleotide additions (B); and the number of different TCRβ amino acid clonotypes (C), estimated for a standard sample size of 48 sequences per gB-8p-specific Vβ10+ TCRβ repertoire. The horizontal lines indicate the median values per age/infection group. A Mann-Whitney test was used for each of the pairwise comparisons between (i) primary and secondary responses in mice primarily challenged as adults, (ii) primary and secondary responses in mice primarily challenged as neonates, (iv) primary responses in adult and neonate mice, and (iv) secondary responses in adult-vaccinated and neonatal-vaccinated mice. Statistical significance was determined at p<0.0125 (*), using Bonferroni correction for multiple pairwise comparisons. The data shown for the neonatal and adult CD8+ T cell responses to primary infection were obtained in previous studies [11], [12] and shown here for comparison with the secondary CD8+ T cell responses.

To determine whether the less-developed T cell repertoires involved in the immune responses to neonatal vaccination are ‘locked-in’ to secondary responses to infection, we examined the clonotypic composition of gB-8p-specific TCRβ repertoires involved in secondary CD8+ T cell responses to HSV-1 infection in adult mice that had been previously vaccinated with VACV-gB either as neonates or as adults. The same Vβ10 gene usage bias associated with primary CD8+ T cell responses to the gB-8p epitope was also observed in both neonatal-vaccinated and adult-vaccinated mice at the peak of the secondary immune responses (Fig. 2A). However, the large inter-mouse variability in Vβ10 gene usage observed in the primary responses [11] in neonatal mice was substantially reduced in the secondary immune responses.

Single-cell sequencing was then used to examine in greater depth the composition of the gB-8p-specific Vβ10+ CD8+ TCRβ repertoires involved in the secondary immune responses. The gB-8p-specific Vβ10+ TCRβ repertoire data are summarized in Table 1 and representative repertoires are shown in Fig. S1, and the gB-8p-specific Vβ10+ CD8+ TCRβ repertoire characteristics quantitatively compared in Fig. S2. It is important to mention that while the primary and secondary responses were elicited by two different pathogens to avoid antibody interference, we previously showed that the clonotypic composition of gB-8p-specific Vβ10+ CD8+ cells in the primary response is similar among a wide range of infections, including VACV-gB and HSV1 [16]. In adult mice, the general characteristics of the gB-8p-specific Vβ10+ CD8+ TCRβ repertoires associated with primary responses were largely preserved in the secondary immune responses (Fig. S2).

**Table 1:** Summary of the gB-8p-specific Vβ10^+^ CD8^+^ TCRβ repertoire data.

	Primary infection^b^		Secondary infection^c^		Resting memory^d^	Primary infection following adoptive transfer^e^	
	Adult^a^	Neonate^a^	Adult-vaccinated	Neonate-vaccinated	Neonate- vaccinated	Primary adult effector	Secondary neonate memory
No. of mice	6	8	9	9	6	6	6
No. of TCRβ sequences across all mice	439	547	539	580	428	485	478
Range of no. of TCRβ sequences per mouse	60–82	52–83	48–71	49–74	65–79	65–86	72–87
Mean no. of TCRβ sequences per mouse	73.2	68.4	59.9	64.4	71.3	80.8	79.7

a These data were obtained in previous studies [11], [12] and are shown here for comparison with the other TCRβ repertoires.

b Obtained at the peak of the CD8+ T cell response following primary VACV-gB challenge.

c Obtained at the peak of the CD8+ T cell response following secondary HSV-1 challenge.

d The resting memory CD8+ T cell population following VACV-gB infection of neonatal mice.

e Obtained at the peak of the CD8+ T cell response to HSV-1 challenge in congenic mice following adoptive transfer of resting memory cells obtained from neonatal mice previously infected with VACV-gB.

In neonatal mice, we have previously reported that the primary response contains a restricted repertoire of T cells that is largely germline encoded [11]. Thus, we expected the secondary response in neonatal-vaccinated mice should also comprise a more restricted subset of these cells. Surprisingly, the secondary response in neonatal-vaccinated mice was significantly more diverse and showed a much higher proportion of TCRβ clonotypes requiring N-additions than the previously reported neonatal primary response [11] (Fig. 2B, C). However, despite this diversification of the gB-8p-specific Vβ10^+^ TCRβ repertoires between primary neonatal responses and secondary responses, TCRβ clonotype diversity remained significantly reduced compared to mice that were primed as adults (Fig. 2C). These results suggest that priming neonatal mice leads to a recall response of intermediate diversity between the neonatal primary response and the ‘normal’ adult secondary response. Thus, we investigated what mechanisms contribute to this partial “locking-in” of the immature neonatal repertoire during secondary responses later in life.

### Neonatal memory CD8+ T cells contribute poorly to a recall response

Since we observe a significant change in the gB-8p-specific CD8+ TCRβ repertoire between the neonatal primary and secondary responses, we set out to identify when this diversification occurred. Firstly, it seems possible that only a subset of the neonatal primary response contributes to the secondary response, and that these cells are selectively the more ‘adult-like’ clonotypes. This selection for adult-like clonotypes could occur either during the contraction phase from the primary response, or during the expansion phase from the memory response to the secondary response. To investigate this, we first analyzed the neonatal resting memory compartment (Fig. S2). Although there was a trend for a more diverse (Fig. S2K, L) and less germline encoded (Fig. S2J) TCRβ repertoire in the resting memory population compared with the neonatal primary response, this was not sufficient to explain our observations for the secondary responses in neonatal-vaccinated mice.

**Figure 3:**
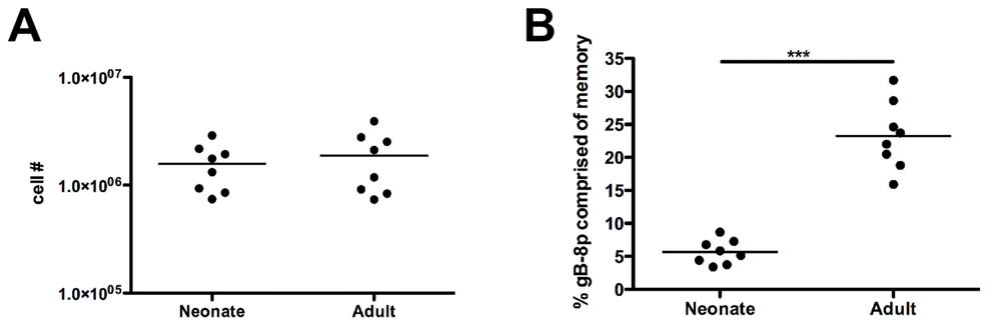
Memory CD8+ T cells from neonatal mice exhibit impaired recall responses. Spleens were harvested from neonatal or adult vaccinated mice (day 42) and equal numbers of gB-8p-specific memory CD8+ T cells (∼2×10^4^ cells) were adoptively transferred into individual naïve congenic (CD45.1) recipient adult mice. Recipient mice were challenged the next day with HSV-1 (1×10^6^ pfu, i.p.). On day 6 post-infection, the spleens of recipient mice were harvested and the (A) absolute number of gB-8p+ CD8+ T cells (i.e. CD45.1 and CD45.2) and (B) the percentage of gB-8p+ CD8+ T cells that were donor-derived memory CD8+ T cells (i.e. CD45.2) were examined. All data is representative of at least 3 experiments, with n = 6–8 mice/group. Results depict mean ± SEM. ***, p<0.001.

A key question is to what extent memory neonatal gB-8p CD8+ T cells participate in a secondary immune response? That is, we would expect neonatal gB-8p memory T cells to be present at higher numbers than adult naive gB-8p T cells, and therefore dominate the recall response. Alternatively, it is possible that the less diverse neonatal T cell clonotypes will be impaired in their ability to compete with a fully developed adult naïve T cell repertoire and thus be underrepresented in the response to a secondary challenge. To examine these possibilities, we adoptively transferred equal numbers of neonatal or adult gB-8p memory CD8+ T cells into different congenic recipient mice (CD45.1) followed by HSV-1 challenge. This allowed us to distinguish between the expansion of naïve (CD45.1) and memory (CD45.2) T cells during the secondary immune response. Donor neonatal and adult gB-8p memory T cells were evaluated at the peak of the recall response following HSV-1 infection, and the proportion of the response contributed by neonatal or adult memory cells was assessed. The magnitudes of the overall response were comparable between the recipients of neonatal memory and adult memory T cells (Fig. 3A). However, approximately 3 fold fewer neonatal memory T cells were observed at the peak of the recall response compared to the adult memory T cells (Fig. 3B). In repeat experiments, we also bled recipient mice at 4 and 6 days post-infection and observed a greater contribution by adult memory CD8+ T cells to the overall response at both time points (Fig. S3). Importantly, these differences were not statistically significant until day 6, suggesting that neonatal memory CD8+ T cell become activated but exhibit an impaired ability to expand and compete in the adult mouse.

**Figure 4:**
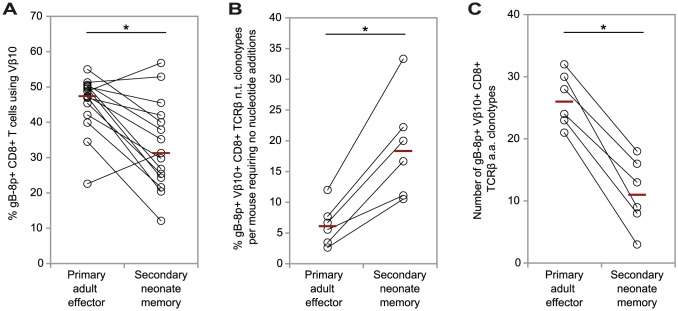
Comparison of the features of the gB-8p-specific TCRβ repertoires between primary adult effector and secondary neonate memory CD8+ T cell populations responding to HSV-1 in congenic mice following the adoptive transfer of resting memory cells from neonatal mice previously infected with VACV-gB. For each recipient mouse the features of the paired primary adult effector and secondary neonate memory CD8+ T cell populations per mouse are shown. The percentage of gB-8p-specific CD8+ T cells that use the Vβ10 gene (A); the percentage of unique gB-8p-specific Vβ10+ TCRβ nucleotide (n.t.) clonotypes requiring no nucleotide additions (B); and the number of different TCRβ amino acid (a.a.) clonotypes (C), estimated for a standard sample size of 48 sequences per gB-8p-specific Vβ10+ TCRβ repertoire are shown. The horizontal lines indicate the median values per group. * p<0.05 (Wilcoxon test).

The reduced contribution of neonatal memory cells compared with adult memory cells in the secondary response raised the possibility that the majority of cells in the secondary recall response in neonatally-vaccinated mice may actually be primary adult CD8+ T cells, rather than neonatal memory cells. To investigate this, we first looked at the clonotypic differences between donor secondary neonate gB-8p-specific memory and the recipient primary adult gB-8p-specific effector CD8+ T cell populations at 6 days post-infection. Although a significantly smaller proportion of the secondary neonatal gB-8p-specific memory T cells used the Vβ10 gene compared with the primary adult gB-8p-specific effector T cells, Vβ10 gene usage was prevalent in most mice (Fig. 4A). Single cell sorting and sequencing of the gB-8p-specific Vβ10^+^ TCRβ clonotypes for these two populations (Table 1; Fig. S1 C, D) revealed that a significantly higher proportion of secondary neonate memory TCRβ clonotypes required no nucleotide additions (Fig. 4B) and the secondary neonate memory TCRβ repertoires were significantly less diverse compared with the primary adult gB-8p-specific effector Vβ10^+^ TCRβ repertoires in the same mouse (Fig. 4C). Furthermore, we verified that the features of the secondary neonate gB-8p-specific memory Vβ10^+^ TCRβ repertoires were indicative of the resting memory population following VACV-gB infection in neonatal mice (Fig. S2 G–R).

To summarize, when we tracked the fate of adoptively transferred neonatal memory cells in the recall response in adult congenic recipients, these neonatal memory TCRβ repertoires maintained similar features to the neonatal memory population. However, the other major contributor to the responding population were the adult primary cells. When we separately analyzed the repertoire of the adult cells contributing to the response to the secondary challenge, we found that they resembled the normal adult secondary response, and were comprised of a significantly higher proportion of TCRβ clonotypes with N-additions, and were significantly more diverse than the neonatal memory cells in the same response. This suggests that the observed ‘diversification’ of the secondary response in neonatal-vaccinated mice arose not because the neonatal repertoire itself was altered, but because the neonatal recall response was so poor, that it was outcompeted by the adult primary response. Therefore, the combination of the narrow neonatal memory and diverse adult primary repertoires led to the observed intermediate level of TCRβ repertoire diversity in the secondary responses in neonatal vaccinated mice.

### The neonatal memory repertoire has lower TCR avidity

**Figure 5:**
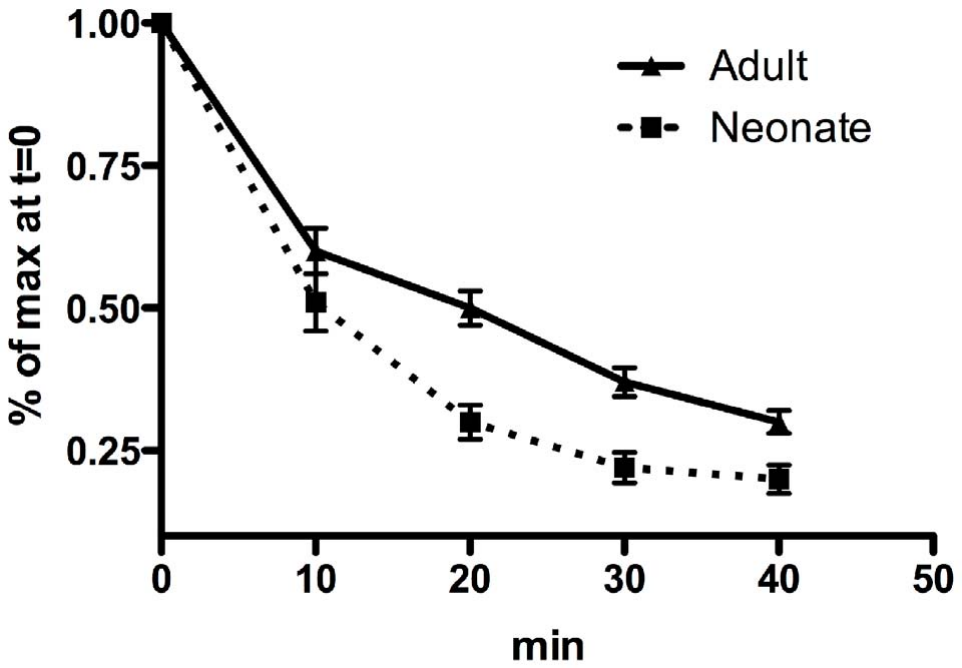
TCR avidity of neonatal and adult memory gB-8p-specific CD8+ T cells. TCR:pMHC dissociation kinetics for splenic CD8+ T cells were also analyzed at 6–7 weeks after VACV-gB infection. Briefly, splenocytes were stained with Kb:gB-8p tetramer for 1 hour at 4°C, washed and incubated in the presence of anti-Kb antibody to prevent rebinding. Depicted here is the relative amount of CD8+ gB-8p+ T cells detected over time ± SEM. Assuming an exponential decay, there is a significant difference (p = 0.003, Mann-Whitney) between the decay rates for neonatal and adult gB-8p-specific CD8+ T cells. All data is representative of at least 2 separate experiments with n = 6–8 mice/group.

Given the less diverse TCR repertoire and poor recall responses exhibited by neonatal memory T cells, we next questioned whether this might be mediated by a neonatal T cell pool is insufficiently broad to select high avidity memory gB-8p CD8+ T cells. This is important since high avidity T cells have been shown to respond more vigorously to infection and kill infected cells faster than low avidity T cells [17]. Our hypothesis was that the responding pool of gB-8p CD8+ T cells in neonates will not include as many ‘best-fit’ T cells and will exhibit much lower TCR avidity than adult gB-8p CD8+ T cells. To test this, TCR:pMHC disassociation kinetics were assessed between neonate and adult memory gB-8p CD8+ T cells. During the steady-state, resting memory phase, neonatal gB-8p CD8+ T cells demonstrated much faster pMHC:TCR off-rates compared to adults (Fig. 5). Collectively, these data suggest that the less diverse neonatal gB-8p memory repertoire undergoes poor recall responses due to lower proportion of high-avidity CD8+ T cells in the memory compartment.

### Neonatal memory CD8+ T cells confer poor immune protection

**Figure 6:**
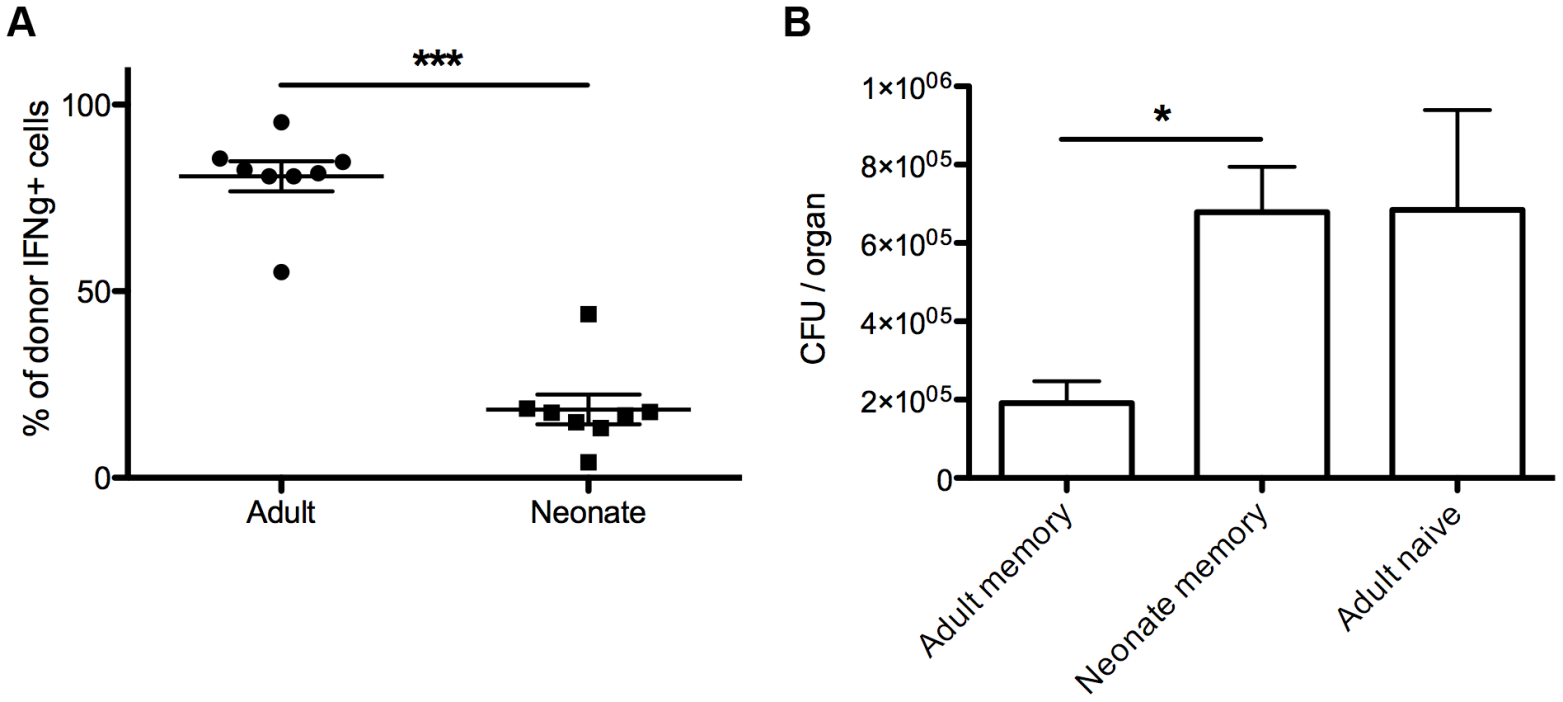
Memory CD8+ T cells from neonatal mice exhibit impaired protective immunity against *Listeria monocytogenes*. (A) Neonatal (CD45.2) and adult (CD45.1) mice were infected with ActA-/- LM-gB (1×10^6^, i.p.) and spleens were harvested from both age groups at 6 weeks post-infection. An equal number of gB-8p-specific memory CD8+ T cells (∼5×10^3^) were co-transferred into the same naïve congenic (Thy1.1) adult recipient and all mice were subsequently infected the next day with HSV-1 (1×10^6^ pfu, i.p.). On day 6 post-infection, spleens were harvested and the relative number of neonatal and adult gB-8p+ memory CD8+ T cells (i.e. CD45.1+ and CD45.2+) were expressed as a percentage of IFNg+ donor cells (Thy1.2). (B) In separate experiments, neonatal and adult mice were again infected with ActA-/- LM-gB (1×10^6^, i.p.) and allowed to transition in to the resting memory phase. At 6 weeks post-infection, neonatal (CD45.2) and adult (CD45.2) memory CD8+ T cells were transferred into separate congenic recipients (CD45.1) and challenged with wild-type LM-gB (5×10^4^, i.v.). On day 3 post-infection, livers were homogenized and the bacterial burden was determined. Results depict mean ± SEM. *, p<0.05.

To validate and broaden the significance of our results, we next asked whether other types of infection also give rise to neonatal memory CD8+ T cells with poor recall efficacy. For these experiments, we infected neonatal and adult mice with an attenuated strain of *Listeria monocytogenes* that expresses the gB-8p peptide (denoted ActA LM-gB). This strain lacks a gene that is required for mobility and is incapable of infecting nearby cells, allowing us to better control for variations in the availability and abundance of antigen and challenge both age groups with the same dose. At six weeks post-infection, we co-transferred equal numbers of neonatal (CD45.2) and adult (CD45.1) gB-specific memory CD8+ T cells into congenically marked recipients (Thy1.1), which were subsequently infected with HSV-1 (1×10^6^ pfu, i.p.). By transferring memory CD8+ T cells from adult or neonatal primed donors into the same host, we could rule out potential environmental differences arising during the response. Importantly, the percentage of neonatal and adult gB-specific memory CD8+ T cells were found to be similar prior to infection, with neonatal donor cells slightly outnumbering adults (68.8%±2.3 vs 30.6%±2.3). At the peak of the response, spleens were harvested and the ratio of neonatal to adult memory CD8+ T cells was calculated. Consistent with our results following vaccinia infection, we again observed a greater contribution of adult memory CD8+ T cells to the secondary response (Fig. 6A). Importantly, this analysis measured the total donor response by IFNg production, and thus included both Vβ10+ and Vβ10- gB-8p specific CD8+ T cells. These findings suggest that limited recall responses are likely a common feature among neonatal memory CD8+ T cells, regardless of how they are initially primed.

Despite the fact that neonatal vaccinated mice exhibited reduced TCR diversity, TCR avidity and lower recall efficiency, a critical remaining question was whether these differences resulted in impaired immune protection. To address this, we compared the ability of neonatal and adult memory CD8+ T cells to clear a high dose infection of recombinant Listeria monocytogenes expressing gB-8p (Lm-gB). We choose to challenge mice with LM-gB instead of HSV-1 for these studies because well-defined sites of infection can be more easily monitored. Neonatal and adult mice were again vaccinated with ActA-/- LM-gB (1×10^6^ cfu, i.p.) and CD8+ T cells were allowed to transition into the memory phase. At 6 weeks post-infection, we transferred the same number of neonatal or adult gB-specific CD8+ T cells into separate recipient mice and challenged both groups with wt LM-gB (5×10^4^ cfu, i.v.). Three days later, the livers of recipient mice were homogenized and the bacterial loads were examined. As shown in Fig. 6B, adult memory CD8+ T cells reduced the bacterial load 3 fold compared to an equivalent number of neonatal memory CD8+ T cells. Indeed, the neonatal memory CD8+ T cells showed no better bacterial control than naïve adult cells (which we have shown contribute significantly to the response in the presence of a neonatal memory response). This data suggests that a suboptimal recall response exhibited by neonatal memory CD8+ T cells could result in reduced immune protection.

## Discussion

The focus of this report was to determine how the composition and responsiveness of the memory CD8+ T cell pool is altered by neonatal vaccination or infection that occurs prior to the diversification of the CD8+ T cell repertoire. Our results demonstrate that the restricted neonatal T cell memory pool induced by early vaccination is in fact comprised of fewer clonotypes that are also of lower avidity than the adult response. However, while vaccination early in life recruits many of these ‘less fit’ clonotypes and allows them to persist in the memory CD8+ T cell compartment, these neonatal memory clonotypes are not efficiently recruited into the proliferative recall response to secondary challenge. In the absence of a strong neonatal memory response mediating early viral control, a robust adult primary response is generated, which ultimately comes to dominate the neonatal memory population. Despite this contribution from the adult repertoire, the secondary response in mice vaccinated as neonates remained significantly restricted, in terms of the diversity of TCR clonotypes, compared with secondary responses in adult-vaccinated mice. These observations describe a situation that is mechanistically similar to the phenomenon of ‘original antigenic sin’, in which prior infections with a related pathogen can “trap” the immune system into responding with less efficient memory clonotypes. However, in this case, the same pathogen may “trap” less efficient clonotypes into the immune reserve simply by priming these T cells during the early stages of development.

One of the most interesting findings of our present study was that there is significantly more recruitment of adult naïve clonotypes into the neonatal secondary response than the adult secondary response. Thus, when neonatal memory CD8+ T cells are faced with competition from a fully-developed adult naïve T cell repertoire, they prove inferior and make a smaller contribution to the overall memory response. Previous reports indicate that new naïve T cells seed the periphery at a relatively constant rate of 1–2×10^6^ cells/day and the number of splenic recent thymic emigrants reach a peak at ∼6 weeks of age [18]. These naïve clonotypes will have also been sculpted by TdT, which should allow for significantly greater opportunities to generate high avidity gB-8p-specific CD8+ T cells. Indeed, our data suggest that more ‘best-fit’ gB-8p-specific CD8+ T cells exist in the adult naïve pool at 6–7 weeks of age compared to those available in the neonate memory pool.

A number of recent reports have examined the role of TdT in generating robust anti-viral CD8+ T cell immunity to acute infections. Mansour Haeryfar et al. showed that the overall magnitude and breadth of the CD8+ T cell responses to influenza and vaccinia virus were reduced in TdT-/- mice and the hierarchy of immunodominant epitopes was altered [19]. The authors proposed that the reshuffling of immunodominant determinants was due to the loss of high affinity clones for some (but not all) viral determinants. In support of this, Kedzierska et. al. showed that the avidity of influenza-specific CD8+ T cells was lower in TdT-/- mice for the NP366 epitope, where the response is public and clonotypically restricted, but not for the PA224 epitope, which elicits a more private and diverse TCR repertoire [20]. Ruckwardt et al. [21] recently reported differences between neonate and adult CD8+ T cell responses to respiratory syncytial virus infection with respect to TCR diversity, functional avidity, precursor frequency and epitope immunodominance hierarchy. However, in terms of the latter, this study suggests that the shifting epitope immunodominance is not associated with TdT. Together, these studies indicate that the relative role of TdT in promoting optimal anti-viral CD8+ T cell immunity may ultimately depend upon the clonal complexity of the T cell response against specific viral determinants being examined.

Although the neonatal repertoire is also comprised of TCRs generated in the absence of TdT, it is important to mention that the neonatal repertoire is potentially even less diverse than adult TdT-/- mice due to lower numbers of T-cells in the neonatal periphery. Based on previous estimates, we would expect ∼2×10^6^ different TCRs (with an average clone size of 10) in adult wild-type mice and ∼1–2×10^5^ different TCRs (with an average clone size of 100) in adult TdT-/- mice [8], [22], [23]. However, there are 10–100 times fewer CD8+ T cells in 7-day old neonatal mice compared to adult mice [24], [25]. Therefore we expect the neonatal repertoire to consist of only a small fraction of the total T cell repertoire that is available in adult TdT-/- mice.

In our report, we elected to prime neonates and adult mice with either an acute virus (VACV-gB) or an attenuated bacterial strain (ActA- LM-gB) so that we could more closely mimic vaccinations and clearly delineate effector and memory CD8+ T cell responses. However, generating sufficiently broad CD8+ T cell repertoires may be even more beneficial in the context of chronic and persistent pathogens. Many of these chronic pathogens (e.g. HIV, HCV) are able to evade the immune response by undergoing a high rate of mutation. Thus, not surprisingly, one key correlate of immune protection against these chronic viral pathogens is diversity in TCR usage [26]–[29]. Responding with a larger number of distinct clonotypes that can recognize multiple epitopes on these pathogens as well as a diverse range of epitope variants has been shown to provide better protection against immune escape. While this has not been rigorously examined in neonatal mice, our prediction would be that the diminished neonatal repertoire would be significantly impaired in limiting the emergence of viral escape mutants.

These results suggest that there are long-term consequences for vaccinations or infections that occur prior to the diversification and maturation of the adult immune system. However, it is important to mention that our results do not rule out the possibility that other developmental factors may contribute to poor neonatal immunity. In this report, we have used TCR analysis as a tool to track neonatal clonotypes, since phenotypic markers alone cannot be used to reliably distinguish naïve and memory CD8+ T cells [30]. Our analysis shows that neonatal clonotypes transition into the adult memory pool, but undergo a limited recall response and confer reduced immunity against a secondary challenge. In regard to these challenge experiments, it is important to point out that pathogen clearance was examined in recipient mice following adoptive transfer of either neonatal or adult memory CD8+ T cells. Thus, only a fraction of the total memory pool is participating in the secondary response. This is an important point since other studies have shown that clearance is dependent upon the number of CD8+ T cells that are adoptively transferred into recipient mice prior to challenge [31]. Under limiting conditions, we observed a statistically significant difference in the ability of neonatal and adult memory CD8+ T cells to clear infection. Importantly, this data does not necessarily indicate that neonatal memory CD8+ T cells are incapable of responding (Fig. 6A clearly shows some contribution of neonatal memory CD8+ T cells to the recall response), but rather that they are less functional compared to adults at the level that was examined. The degree of immune protection by neonatal memory CD8+ T cells will likely vary with the number and type of memory cells that are generated [32]. Given that neonatal T cell clonotypes do in fact gain access to the memory pool in adults, it is now imperative that we fully understand how neonatal memory CD8+ T cells differ than adult memory CD8+ T cells at the cellular and molecular level. These studies would be especially important to consider in the context of tissue resident memory T cells, or the long-lived population of T cells that remain detached at the peripheral sites of initial pathogen encounter (i.e. lung, skin, gut, etc). Knowledge from these studies will provide us with a solid platform to understand how infections early in life may impact the development of T-cell mediated diseases in adulthood, and also to guide rational design of vaccines that can be safely administered to neonates.

## Materials and Methods

### Mice

C57BL/6 (H-2b) and B6-LY5.2/Cr (H-2b) mice were purchased from NCI (Frederick, MD) and Thy1.1 mice (B6.PL-Thy1a/CyJ) were obtained from The Jackson Laboratory (Bar Harbor, Maine). All mice were maintained under pathogen-free conditions in the animal facility at either the University of Arizona or Cornell University. Pregnant mice were individually housed and monitored daily for births. Neonatal mice were used at 7 days of age. Adult mice were obtained from commercial vendors and used at 2–3 months of age.

### Ethics statement

All animal experiments were conducted by guidelines set by the University of Arizona Institutional Animal Care and Use Committee (IACUC), under the University of Arizona approved animal protocol #08-059, and in accordance with the U.S. Animal Welfare Act. Recombinant vaccinia virus (VACV) expressing the MHC class I-restricted CTL epitope HSV gB_498–505_ (SSIEFARL, gB-8p in the text), designated VACV-gB, was generously provided by Dr. S.S. Tevethia (Pennsylvania State University of College Medicine, PA). VACV-gB viral stocks were propagated and quantified in 143B cells. HSV-1 strain 17 was obtained from Dr. D.J. McGeoch (University of Glasgow, Scotland, U.K.), cloned as a syn+ variant and tittered on Vero cells in our laboratory as previously described [33], [34]. Neonatal and adult mice were intraperitoneally infected with either 2×10^1^ or 2×10^5^ PFU, respectively. Recombinant strains of *Listeria monocytogenes* expressing the gB-8p epitope, designated Lm-gB or ΔActA Lm-gB, were provided by Dr. Sing Sing Way (Cincinnati Children's Hospital Medical Center, OH) and have been previously described [35]. Prior to infection, the bacteria were grown to log phase (OD_600_ 0.1), and mice were either immunized with ΔActA Lm-gB (1×10^6^ CFU, i.p.) or challenged with LM-gB (5×10^4^ CFU i.v.) in 100 ul of PBS.

### Reagents and flow cytofluorometric (FCM) analysis

The gB-8p:K^b^ tetramer was obtained from the National Institutes of Health Tetramer Core Facility (Emory University, Atlanta, GA). mAbs anti-CD8α (clone 53–6.7), anti-CD4 (RM4-5), anti-CD11a (2D7), anti-Vβ10 (B21.5), anti-Vβ8 (F23.1), anti-CD45.2 (104) were purchased from commercial sources. FCM data was acquired on the custom-made FACS LSRII instrument equipped with four lasers, using Diva software (BD Biosciences), and analysis was performed using FloJo software (Treestar).

### TCR avidity assays

To evaluate the degree of TCR avidity, the relative off-rates were determined by a tetramer decay assay. For this, splenocytes were stained with anti-CD8α and gB-8p:Kb tetramer for 1 hour at 4°C. These cells were then washed and incubated in the presence of saturating amounts of anti-K^b^ antibody (AF6; Biolegend) at room temperature to prevent rebinding. At various times, cells were removed, placed in fixation buffer and the amount of gB-8p:Kb tetramer remaining on the surface was quantified by flow cytometry. These measurements were expressed as a percentage relative to tetramer staining at time t = 0.

### Single-cell sorting and RT-PCR

Splenocytes were harvested at indicated timepoints following infection. CD8+ T cells were isolated using positive immunomagnetic selection (Miltenyi Biotec, Auburn, CA) and CD8+CD4-gB-8p:K^b^+ Vβ10+ lymphocytes were individually sorted using the FACSAria cell sorter system (BD Biosciences) as previously described [12], [35]. Control wells without sorted cells were included on every plate to identify any possible contamination. cDNA synthesis, PCR amplification and sequencing of individual Vβ10 transcripts were performed exactly as previously described [12], [35].

### TCRβ repertoire analysis

The gB-8p-specific CD8^+^ TCRβ repertoires were characterized by sequentially aligning each TCRβ sequence with the Vβ10 (TRBV4 in IMGT nomenclature) gene, followed by the best-match Jβ gene and the best-match Dβ gene. This analysis was done using the IMGT reference alleles for the *Mus musculus* TRB genes [36]. The CDR3β sequence was then identified between, and inclusive of, the conserved cysteine in the Vβ-region and the conserved phenylalanine in the Jβ-region.

The diversities of the CD8^+^ TCRβ repertoires specific for the gB-8p-epitope in each mouse were evaluated using two different measures of diversity, the number of different TCRβ amino acid sequence clonotypes and Simpson's diversity index [37]. Simpson's diversity index accounts for both the variety of amino acid sequence clonotypes and their clone sizes, and ranges in value from 0 (minimal diversity) to 1 (maximal diversity). To account for differences in the sizes of the TCRβ repertoire samples, TCRβ repertoire diversity was estimated as the median value of 10,000 random draws of subsamples of 48 TCRβ sequences from the total TCRβ repertoire sample [37]. The diversity analysis was performed using Matlab (The Mathworks, Natick, MA).

### *In vivo* bacterial clearance

To evaluate immune protection, 5×10^4^ CFU of wt Lm-gB was administered intravenously as previously described [38]. On day 3 post-infection, livers were harvested into sterile PBS and weighed. Tissues were homogenized mechanically using a Tissue-Tearor electric homogenizer (BioSpec Products, Bartlesville, OK). Serial dilutions were made in sterile PBS and plated onto BHI agar. Plates were incubated overnight at 37°C. The log_10_ CFU/g of tissue was calculated as: log_10_ [(CFU/dilution factor)×(organ weight+homogenate volume)/organ weight)].

### Statistical analysis

TCRβ repertoire features of the endogenous CD8+ T cell responses were compared using a Mann-Whitney test for all pairwise comparisons between age/infection groups, with Bonferroni correction for multiple pairwise comparisons. TCRβ repertoire features of the recipient and adoptively transferred CD8+ T cell populations were compared using a Wilcoxon text. For the tetramer decay assay results, exponential decay rates for individual mice were compared between neonatal and adult CD8+ T cells using a Mann-Whitney test. All statistical analyses were performed using GraphPad Prism software (GraphPad Software Inc, San Diego, CA).

## Supporting Information

Figure S1 **Examples of the gB-8p-specific Vβ10+ TCRβ repertoires.** Examples of the gB-8p-specific Vβ10+ TCRβ repertoires involved in the secondary CD8+ T cell responses to HSV-1 infection in mice that were previously challenged with VACV-gB either as neonates (A) or as young adults (B). The gB-8p-specific Vβ10+ TCRβ repertoires involved in CD8+ T cell responses to HSV-1 infection in congenic mice that were derived from the recipient primary adult effector population (C) and the donor neonatal secondary memory population (D). A more detailed comparison of the TCR repertoire features between groups is shown in Figures 2, 4, and S2.
(EPS)

Figure S2 **Summary of the features of the gB-8p-specific TCRβ repertoires.** The features of the gB-8p-specific Vβ10+ TCRβ repertoires for primary and secondary CD8+ T cell responses to VACV-gB and HSV-1 infection, respectively, for mice that were initially vaccinated as neonates or as adults (A–F) and for the resting memory population following VACV-gB infection in neonatal mice (G–L). The features of the gB-8p-specific TCRβ repertoires for the primary adult effector and secondary neonate memory CD8+ T cell populations responding to HSV-1 in congenic mice following the adoptive transfer of resting memory cells from neonatal mice previously infected with VACV-gB (M-R). Shown are the percentage of TCRβ amino acid (a.a.) clonotypes pooled across all mice per age/infection group that have a particular CDR3 length (evaluated inclusive of the conserved cysteine in the Vβ-region and the conserved phenylalanine in the Jβ-region)(A, G, M), and use a particular Jβ gene (B, H, M), the percentage of TCRβ amino acid clonotypes per mouse that feature a tryptophan-glycine (WG) doublet in CDR3β positions 6 and 7 (which correspond to CDR3 positions 3 and 4 using the Chothia definition [39])(C, I, O), the percentage of TCRβ nucleotide (n.t.) clonotypes per mouse that require no nucleotide additions (D, J, P), the number of different TCRβ amino acid clonotypes (E, K, Q) and the Simpson's diversity index (F, L, R). The diversity measures were estimated for a standard sample size of 48 sequences per gB-8p-specific Vβ10+ TCRβ repertoire. The horizontal lines in Panels C-R indicate the median values per age/infection group. (C–F) * p<0.0125 (Mann-Whitney test with Bonferroni correction for multiple pairwise comparisons between (i) primary and secondary responses in mice primarily challenged as adults, (ii) primary and secondary responses in mice primarily challenged as neonates, (iii) primary responses in adult and neonate mice, and (iv) secondary responses in adult-vaccinated and neonatal-vaccinated mice). (O–R) # p<0.05 (Wilcoxon test). The data shown for the neonatal and adult CD8+ T cell responses to primary infection were obtained in previous studies [11], [12] and shown here for comparison with the other TCRβ repertoires.
(EPS)

Figure S3 **Neonatal memory CD8+ T cell undergo limited expansion in adult recipient mice.** Neonatal and adult memory CD8+ T cells from mice previously vaccinated with VACV-gB were adoptively transferred into adult congenic recipients and challenged the next day with HSV-1 (1×10^6^ pfu, i.p.). On days 4 and 6, recipient mice were bled and the relative amounts of neonatal or adult memory cells were examined and expressed as a percentage of the total gB-8p+CD8+ T cell response (n = 8 mice/age group/time point). Results depict mean ± SEM, n = 8 mice per group, *, p<0.05.
(TIFF)
